# Primary care management strategies and COVID-19 related hospitalization: a population-based study

**DOI:** 10.1093/eurpub/ckac129.537

**Published:** 2022-10-25

**Authors:** L Palandri, A Ferrari, A Serafini, S Riccomi, G Ugolini, PP Kurotschka, F Bellelli, M Morandi, MS Padula, E Righi

**Affiliations:** 1 Department of Biomedical, Metabolic and Neural Sciences, University of Modena and Reggio Emilia, Modena, Italy; 2 Department of Primary Care, Local Health Unit of Modena, Modena, Italy; 3 Department of Medical Science and Public Health, University of Wuerzburg, Wuerzburg, Germany

## Abstract

**Background:**

Due to SARS-CoV-2 rapid mutations, the ending of the pandemic is still proceeding at a slow pace and there is the need to strengthen and invest in health systems that avoid hospital overload and its consequences on patients’ health. Most symptomatic infections have mild to moderate respiratory symptoms and patients are managed in the context of primary care. In Italy, literature on COVID-19 outpatients management by general practitioners (GPs) is scarce. This study explores the effect of GP active care and monitoring on COVID-19-related hospitalization in patients in the province of Modena (Italy) and investigates the possible determinants of GP's management.

**Methods:**

This is a retrospective cohort study of SARS-CoV-2 infected adult outpatients managed by their GPs from March 2020 to April 2021 in the province of Modena (Italy). Data on GPs’ characteristics, management strategies (visits and remote monitoring), patients’ socio-demographic characteristics, and hospitalization were extracted from the GP's electronic medical records and were analyzed using descriptive statistics and multiple logistic regression.

**Results:**

46 GPs agreed to participate, and 5340 patients were included in the analyses. 3014 (56%) patients received active daily remote monitoring and 840 (16%) were visited at home. Remote monitoring and home visits were both associated with a reduction of the probability of hospitalization rate of approximately 50% (respectively OR:0.52, 95%CI:0.33, 0.80 and OR:0.50, 95%CI;0.33, 0.78). Preliminary analysis of determinants showed that GPs’ patient load, setting, age, and sex were significantly associated with management strategies.

**Conclusions:**

Active monitoring performed by GPs was effective in reducing the probability of hospitalization. Primary and hospital care integration can be effective for COVID-19 management. Studies on GPs’ characteristics and patient load and their effect on their ability to care for patients are needed.

**Key messages:**

• Active remote monitoring and visits performed by Italian general practitioners effectively reduced hospitalization for COVID-19.

• Primary and hospital care integration can be effective for COVID-19 management.

